# Public health benefits of zero-emission electric power generation in Virginia

**DOI:** 10.1016/j.heliyon.2023.e20198

**Published:** 2023-09-15

**Authors:** Luis E. Ortiz, Reilly Stiles, Sophia Whitaker, Edward Maibach, James Kinter, Lucas Henneman, Jenna Krall, Paul Bubbosh, Benjamin Cash

**Affiliations:** a Virginia Climate Center, George Mason University, Fairfax, VA, USA; b Department of Atmospheric, Oceanic, and Earth Sciences, George Mason University, Fairfax, VA, USA; c Center for Ocean, Land and Atmosphere Studies, George Mason University, Fairfax, VA, USA; d Center for Climate Change Communication, George Mason University, Fairfax, VA, USA; e Department of Civil, Environmental And Infrastructure Engineering, George Mason University, Fairfax, VA, USA; f Department of Global and Community Health, George Mason University, Fairfax, VA, USA; g Schar School of Policy and Government, George Mason University, Fairfax, VA, USA

## Abstract

Curbing the worst impacts of global climate change will require rapidly transitioning away from fossil fuel across all sectors of the economy. This transition will also yield substantial co-benefits, as fossil fuel combustion releases harmful pollutants into the air. In this article, we present an analysis of the co-benefits to health and health-care costs related from decarbonization of the power sector, using the Virginia Clean Economy Act (VCEA) as a case study. Using a model that combines a source-response matrix approach to pollutant concentration modelling tied to health impact functions, our analysis shows that, by 2045, the VCEA will save up to 32 lives per year across the state, and avoid up to $355 million per year in health-related costs. Fossil-fuel free generation will also help the most disadvantaged communities, as counties in the highest poverty rate quintile also avoid the most pollutant-related deaths.

## Highlights

• Zero-emission electric power generation leads to 14 to 32 fewer deaths per year.
• Economic benefits range between $140 and $355 million USD in avoided health costs.
• The poorest counties in Virginia are projected to see the greatest benefits.

## 1 Introduction

Curbing the worst impacts of global climate change will require a rapid transition from fossil fuel power generation to clean (i.e., non-polluting) technologies across all sectors of the economy. Throughout the U.S., electric power generation accounted for 25% of all carbon emissions in 2020 [1]. This transition away from fossil fuels can yield benefits beyond mitigating climate change. In addition to reducing emission of heat-trapping greenhouse gases (GHG), the combustion processes involved in electric power generation release other pollutants like particulate matter (e.g., PM_2.5_ and PM_10_), sulfur dioxide (SO_2_) and nitrogen oxides (NO_x_) into the air. Studies show that both acute and chronic exposure to these pollutants contributes to cardiovascular, respiratory, and cerebrovascular mortality [[2], [3], [4]]. Exposure to these pollutants also leads to increased hospitalization rates for respiratory and cardiovascular disease [[5], [6], [7]]. Of these pollutants, PM_2.5_ has been strongly linked to increased mortality and morbidity risks, with an US Environmental Protection Agency (US EPA) Integrated Science Assessment (ISA) finding strong scientific support for a likely causal link between exposure to PM_2.5_ and adverse health effects [8].

In 2020, the Commonwealth of Virginia enacted the Virginia Clean Economy Act (VCEA) [9]. The VCEA mandates, among other things, that all electric power generation within the state of Virginia shifts away from fossil fuels by 2045. Virginia has historically relied on coal to meet most of the state's electricity demand. In 2020, Virginia generated about 82% of its total electric energy consumption, importing the rest from neighboring states. Carbon emissions in the state peaked in the year 2005, when they reached nearly 130 million metric tons of CO_2_ [10]. Since then, despite a growing demand for electricity, CO_2_ emissions have dropped to 98 million metric tons of CO_2_, a 20-year low, mostly due to a transition from coal to natural gas as the leading source of electricity generation. Natural gas has become the dominant fuel for electric generation in the state, powering 57% of the total fuel mix and nuclear energy providing 30%. This increase has mostly come due to phasing out of coal power, although there have been minor increases in generation from renewable sources like solar, which now provides approximately 4% of in-state generation [11].

The recent statewide adoption of natural gas in place of most of the state's coal power plants has led to large reductions in both greenhouse gas emissions and traditional air pollutants such as particulate matter, SO2, and NOx. Short-term advantages of this energy transition, however, do not account for potential and uncertain disbenefits including 1) unaccounted-for methane and other volatile organic compound emissions and 2) economic “lock-in” associated with long lifespans of natural gas power plants [12,13]. Nevertheless, the shift away from fossil-fuel emissions has already yielded significant benefits for Virginia and the entire Mid-Atlantic region. A study by Millstein et al. [14] found that increased wind and solar generation in the Mid-Atlantic was responsible for $300 million in benefits due to avoided deaths and morbidity related to air quality improvements. Due to reduced ambient PM_2.5_ and ozone.

Analyses of multi-sectorial decarbonization policies in the US have been performed at scales ranging a single state to country-wide. Driscoll et al. (2015) [15] modeled potential benefits in O_3_ and PM_2.5_ concentrations from a series of policies including the EPA Clean Power Plan using. They accomplished this by simulating pollutant transport with the Community Multi-scale Air Quality Modeling System (CMAQ) at 12 km resolution, and estimating state-level health co-benefits using the Environmental Benefits Mapping and Analysis Program (BenMap). Similar analyses have quantified health co-benefits of state-level decarbonization policies. For example, Zhu et al. (2022) [16] showed, the census tract level, that decarbonization would improve air quality for vulnerable populations using CMAQ and BenMap. Similarly, Johnson et al. (2020) [17] analyzed New York City's 80 × 50 decarbonization strategy across the energy, energy, and transportation sectors using these same modeling technologies at the single-city scale.

We present an analysis of the health and health cost benefits from transitioning to fossil fuel-free electric generation in Virginia following the recently enacted Virginia Clean Economy Act (VCEA). Our analysis uses the US Environmental Protection Agency's (US EPA) COBRA model to investigate, at the county level, the health and health-cost benefits of decarbonization of the power sector in the state. The COBRA model uses a reduced-complexity air quality model in combination with health impact functions to estimate the health benefits of reduced air pollutants due to decarbonization in the power sector. To the authors' knowledge, this is the first such analysis of the VCEA law, one of only 11 state-level legislations mandating decarbonization throughout the 21st century. These impacts include avoided deaths and their related costs, as well as the cost of missed workdays and avoided hospitalizations due to cardiovascular and respiratory illness. We focus on these health adverse effects as representative of co-benefits to lives, health, and livelihoods, although other benefits from reduced respiratory and cardiovascular disease exacerbations may also exist from reduced exposures to fossil fuel combustion emissions.

## 2 Materials and methods

This section describes our methods to quantify the health benefits of decarbonization of Virginia's power sector. Our analysis strategy relied on modelling the decarbonization pathway of the VCEA, which mandates a complete transition away from fossil fuels between 2045 and 2050. Our modelling approach uses a combined reduced-complexity air quality risk assessment model that estimates adverse health outcome reductions and costs of air pollution emissions reductions.

### 2.1 COBRA

Our analysis was completed by using the US Environmental Protection Agency's CO-Benefits Risk Assessment Health Impacts Screening and Mapping Tool (COBRA). COBRA links a combination of models to estimate impacts of modifying emissions on ambient pollution and health. To accomplish this, it uses extensive data on U.S. populations and fossil fuel emissions, as well as projections of these factors for years in the near future. The model estimates economic and health benefits based on fossil fuel combustion emissions modifications, which are then converted into approximate changes in ambient PM_2.5_ concentrations [18]. COBRA includes baseline data for the years 2016, 2023, & 2028, all of which include baseline emissions, populations for all U.S. counties.

Historical emissions data for the 2016 baseline year includes actual historical records from the EPA 2016 version 1 Air Emissions Modeling Platform, whereas 2023 and 2028 use forecasts based on current emissions and population trends. Emissions sources include fuel combustion from utility and industrial sectors, materials processing, transportation, and natural sources. Historical and future population data is bundled with COBRA and obtained from the Woods and Pool (2015) [19] population data. This population dataset uses historic county populations from the US Census, and models future county-level population using historical economic trends, net-migration and birth and natural cause mortality rates. Moreover, population is partitioned by age and sex. The COBRA tool has been used in studies quantifying the benefits to children's health of the US Regional Gas Initiative [20], an assessment of actual benefits of clean coal [21], and impacts of prescribed burning on vulnerable communities [22].

Pollutant concentrations in COBRA are estimated using a Source-Receptor Matrix (SRM) reduced complexity approach. Rather than estimate concentrations by dynamically modeling atmospheric transport, chemical reaction, and deposition processes, this approach pre-computes, for each administrative boundary (e.g., county) of interest, the relationship between all pollutant sources and concentrations. Concentrations are estimated for a hypothetical receptor located at the area centroid of each administrative boundary. To estimate relationships between emissions and concentrations, or transfer coefficients, at the hypothetical receptor, COBRA uses results from a Gaussian dispersion model. SRM approaches have been used to study industrial emissions in Chile [23] and power-sector pollution in Georgia [24], among others. Levy et al. (2003) [24] also found that results from SRM methods compared favorably to more a more complex Lagrangian puff model (CALPUFF). Gilmore et al. [25] compared three reduced complexity models in the context of evaluating the social cost ambient pollutants, finding that in spite of significant differences in their formulation, loss of accuracy when compared to complex models was modest. More recently, Henneman et al. [26] found a the normalized mean error of 28% in a reduced complexity inverse source-receptor calculation when compared to chemical transport model at the annual individual power plant scale, with performance reductions from distance and upwind from sources.

Health impact functions in COBRA are based on dose-response relationships reported in the scientific literature that link pollutant concentrations to health outcomes with either linear or log-linear models. In this study, we consider PM_2.5_ impacts, for which evidence is most strongly linked to mortality and other health impacts [8]. Where more than one reported model exists for a particular pollutant-health impact function, these are combined using a mixed effects model following the methods from DerSimonian and Laird (1986) [27]. In these cases, outputs include a low and high estimate to account for varying health impact functions. Table 1 details the source of the concentration-response functions used for health impacts. Mortality impacts consider long-term exposures, while hospital admissions and work loss days results concentration-response functions are based on short term exposure.

**Table 1:** Concentration-response functions used to define health impacts in this study.

Health impact function	Citation
Mortality, PM_2.5_	Krewski et al., n.d., Lepeule et al., 2012, Woodruff et al., 1997 [[28], [29], [30]]
Hospital admissions	(Babin et al., 2007; Bell et al., 2008; Kloog et al., 2012; Moolgavkar 2000a, 2000b; Peng et al., 2009; Sheppard 2003; Zanobetti et al., 2009 [[31], [32], [33], [34], [35], [36], [37], [38]]
Work Loss Days	Ostro 1987 [39]

Meanwhile, costs linked to these health impacts are assessed differently for mortality and health-care costs. A value of statistical lives approach is followed to quantify the benefit of avoided deaths, with a value of $9.5 million for 2016. This value is based on multiple studies and adopted as EPA policy [40]. Mortality impacts are spread over a 20-year lag for a particular change in concentrations, although these are counted on a single year. Because of the use of this 20-year lag, valuation of mortality risk avoidance uses a discount rate to account for temporal preferences in benefits (i.e., most people would prefer risk reductions now rather than a later date). This preference is quantified by discounting benefits received at future dates. Although there is no currently accepted value for this discount rate, EPA and the US Office of Management and Budget (OMB) suggest values of 3% and 7%, respectively. In our analysis, we run simulations with both discount rates in order to include a broader range of outcomes.

### 2.2 Scenario configuration

**Fig. 1:**
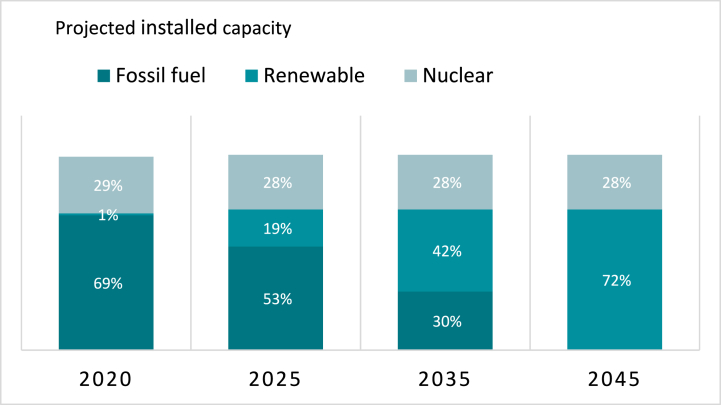
Current decarbonization pathway defined by the Virginia Clean Economy Act of 2020. Source: Virginia Energy Plan (2022).

We quantify the health and health cost benefits of shifting the power sector from fossil fuel combustion by designing a series of COBRA runs that follow the pathway mandated by the VCEA. Our analysis geographic unit is the county. We accomplish this by modifying emissions factors at the county level to follow the share of power generation provided by fossil fuels as shown in Fig. 1. Rather than assume a sequence of individual power plant retirements, our analysis assumes a uniform reduction throughout the state. We assume fossil-fuel use reductions only within the state of Virginia, keeping emissions outside the state constant. Because of this assumption, any remaining health impacts in-state are from out-of-state sources as well as non-utility generation emissions (e.g., transportation and industrial sectors). Simulations were performed for the years shown in the Virginia Energy Plan [11], which include 2025, 2035, and 2045 (Fig. 1). We added an additional year, 2030, to include additional information at a medium-term horizon.

Emissions in COBRA are organized in three tiers, with tier 1 encapsulating the processes that created the emissions, such as fuel combustion, metal processing, and highway vehicles. Tier 2 represents the fossil fuel type burned (e.g., coal or natural gas), and Tier 3 representing specific fuels (e.g., bituminous coal). To emulate the VCEA, we modify emissions at the Tier 1 level, reducing emissions from all fossil fuels used in utility-scale generation.

Health impacts estimates rely on demographic data for each county. Population total, broken down by age group, was sourced from the Woods & Poole database, which includes both historical records as well as forecasts up to the year 2050 [19]. Model outputs (health outcomes and costs) are reported at the county level, which is the finest boundary size available in COBRA. The health impacts considered in this study include all-cause mortality [[28], [29], [30]], hospital admissions [[31], [32], [33], [34], [35], [36], [37], [38]], and work loss days [39]. In addition, we include the monetized impacts of avoided health impacts.

Finally, we study the impact of county-level socioeconomic disparities on avoided mortality. To accomplish this, we use data describing the percent of the population with income below 150% the national poverty level sourced from the Center for Disease Control (CDC) Social Vulnerability Index [41], which is estimated based on data from the American Community Survey [42]. We use the most recent 3-year estimate, aggregated to county boundaries to relate locations that would most benefit from removal of fossil fuel pollution.

## 3 Results

### 3.1 Emissions reductions

**Fig. 2:**
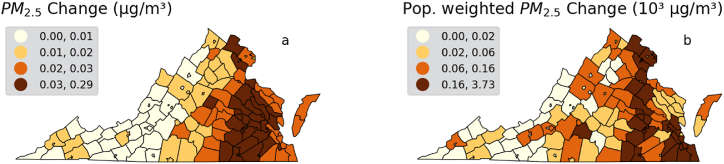
Ambient PM_2.5_ concentration reduction (in μg/m^3^) per year from 100% fossil-fuel free power generation in total units (a) and population weighted units (b).

**Fig. 3:**
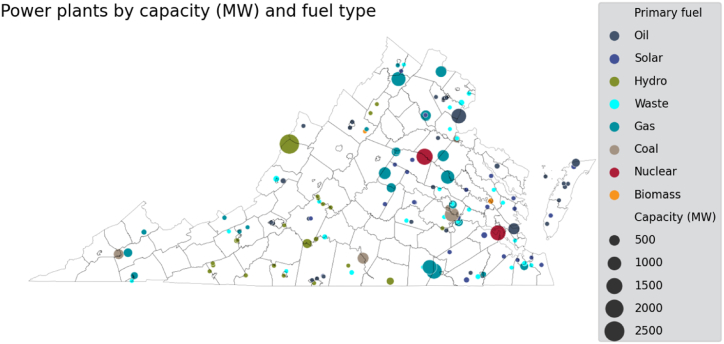
Power plants in Virginia by primary fuel type and installed capacity. Data obtained from the Global Power Plant Database, version 1.3.

Elimination of fossil fuel power from Virginia will prevent the release of large quantities of the harmful pollutant PM_2.5_ and its gaseous precursors into the air, as shown in Fig. 2 (left). The largest exposure reductions will occur throughout the eastern part of the state, coinciding with the largest concentrations of fossil fuel plants (Fig. 3) and largest population centers in the state. The largest reductions in PM_2.5_ will be in highly populated areas like Fairfax County and the Richmond metropolitan area reaching up to 0.29 μg/m^3^ annually, impacting nearly 1.4 million people in these two locations alone (Fig. 2, right). The remaining ambient PM_2.5_ in 2045 will come from sources like transportation, industry, and out-of-state fossil-fuel generation – unless those sources of pollution are also reduced.

### 3.2 Health benefits

**Fig. 4:**
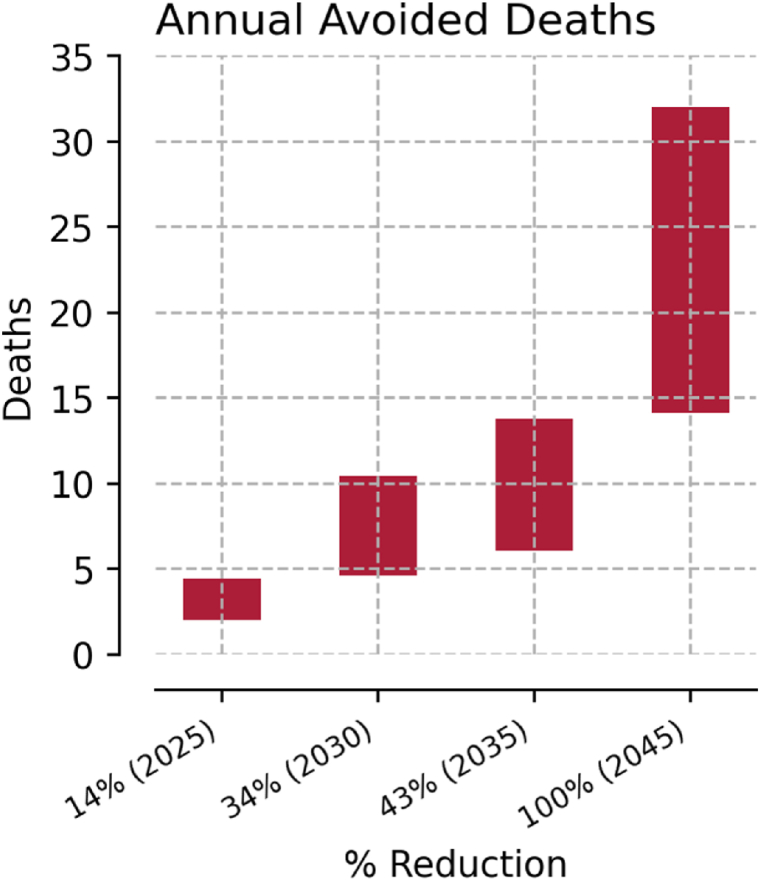
Total yearly avoided deaths by percent reduction in electric generation from fossil fuels. The bars show the low and high estimates in outcomes by percent reduction in fossil-fueled emissions.

Reducing emissions from fossil-fuel power plants will reduce mortality. Benefits will increase throughout the assumed phase-out period, providing both immediate and long-term benefits to Virginia's communities. By 2025, if emissions from electricity production have been reduced by 14%, the annual number of avoided deaths is projected to be between 2.0 and 4.4. By 2045, if emissions have been reduced by 100%, the annual number of avoided deaths is projected to be between 14 and 32 (Fig. 4). Although the range between the high and low estimate increases as the amount of fossil fuel decreases, even the lowest avoided mortality estimate in 2045 is larger than the high-end estimate for 2035.

**Fig. 5:**
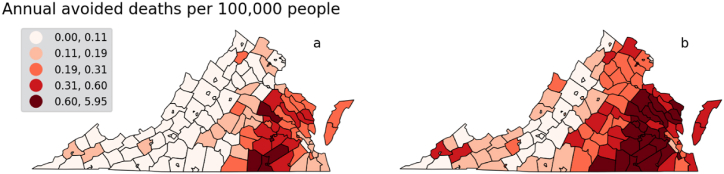
Low (a) and high (b) estimates of annual avoided deaths per 100,000 people by county.

At the county-level, avoided mortality rates are higher throughout the eastern Virginia than western Virginia (Fig. 5). This region includes the county with the largest remaining coal plant in Virginia, Chesterfield County, which can expect to avoid between 15 and 36 deaths per decade after 100% phase-out of fossil fuels. When normalized by county population, the impacts on less populous regions become apparent. The top examples include Emporia and Brunswick counties, which could avoid 26–59 and 20–45 deaths per 100,000 residents. In spite of having relatively low populations to larger population centers in the state at just over 5000, Emporia's relatively high population density (762 people per square mile) and proximity to large gas generation increase relative exposure levels. Meanwhile, Brunswick County houses one of the state's largest gas-powered plants at 1.4 MW capacity, leading to high relative exposures in spite of low population density (28 people per square mile).

**Fig. 6:**
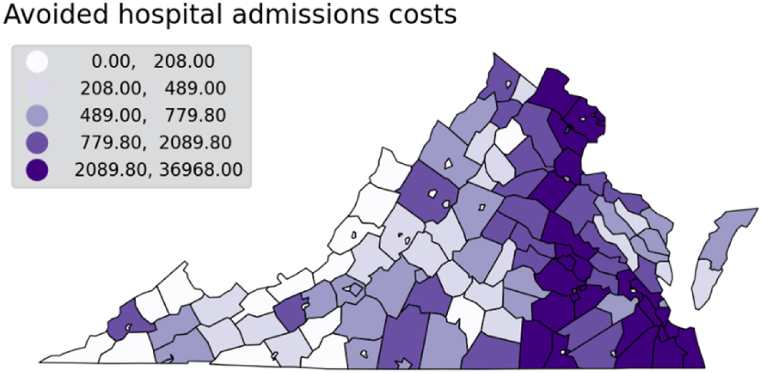
Estimated annual avoided cost of hospitalizations (respiratory and cardiovascular causes) due to PM_2.5_ exposure.

**Fig. 7:**
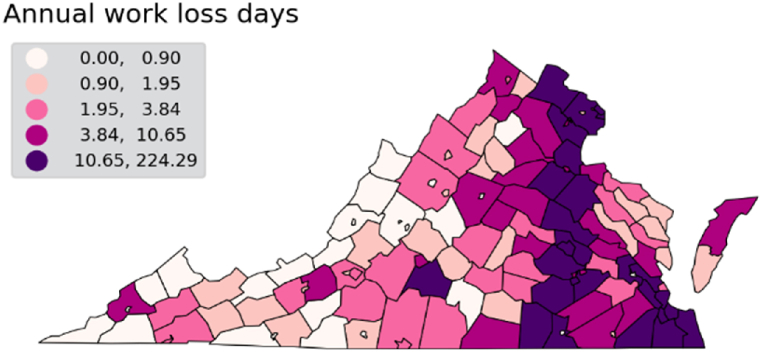
Estimated annual avoided work loss days per 100 people by county.

The health benefits of fossil fuel phase-out are not limited to avoided deaths from respiratory and cardiovascular diseases. Costs of hospitalization admissions due to exposure to PM_2.5_ are highest in the eastern side of the state, reaching approximately $37,000 in the highest quintile (Fig. 6). Exposure to pollutants also impact workers and the workforce, measured in COBRA through a health impacts function relating PM_2.5_ concentration changes to work loss days [39]. Results show the majority of work loss days occur in highly populated counties through the eastern half of Virginia, and where a large amount of installed generation capacity is located. Values of work loss days in the top quintile start at 11 and can go as high as 224 work loss days per 100 people per year (Fig. 7). We note that results for work loss days only include the population aged 18–64, which were part of the Ostro 1987 study. The share of the workforce age 65 and older has increased and is projected to keep increasing as total population grows older, having reached close to 16% in 2010 (US Bureau of Labor Statistics). However, although older adults have higher risks of being impacted by air pollutants, there are no dose-response studies linking work loss days in that population.

We also present monetized benefits of the overall health costs of reaching 0% fossil fuel generation. Our analysis using the COBRA tool includes a range of health benefits from avoided emissions. These impacts include work loss days and mortality-related benefits, as well as those related to reduced incidence in respiratory and cardiovascular disease-linked hospitalizations.

**Fig. 8:**
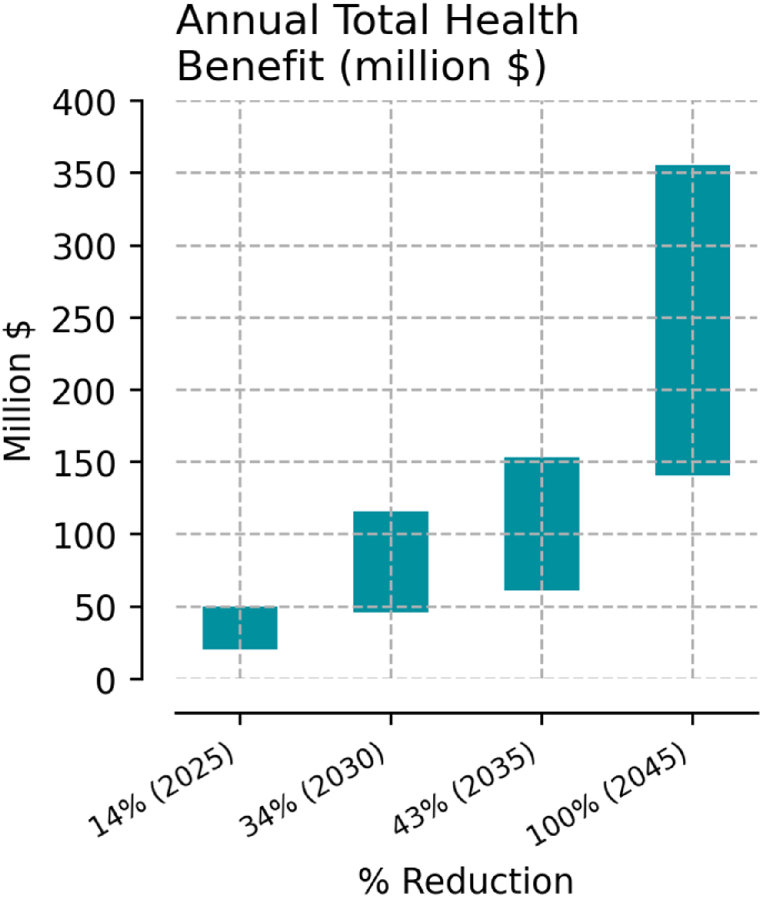
Total yearly economic benefit from avoided health costs and mortality. The bars show the low and high estimates in outcomes by % reduction in fossil-fueled emissions.

**Fig. 9:**
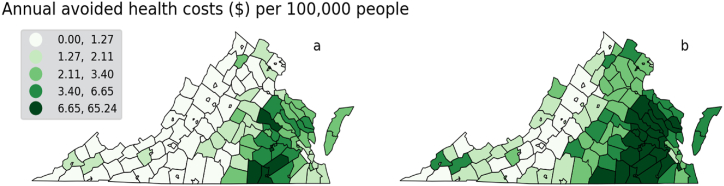
Low (a) and high (b) estimates of annual avoided health costs (million $).

Results show that economic benefits of avoided health impacts begin to accrue at modest levels of fossil fuel phase-out. At 13.9% reduction, estimated total benefits range between 20 and 50 million dollars per year (Fig. 8). With fully non-fossil fuel generation, total avoided health cost lies between $140 and $355 million annually. The increase in benefits mirrors results from mortality data, as the economic benefit of avoided mortality is the strongest driver of total economic benefits. This result is also reflected at the county level, where a combination of proximity to fossil fuel power plants and population places the highest economic benefits in counties along the Eastern half of the state. These include highly populated counties like Fairfax and Prince William in Northern Virginia, as well as Henrico County in the Richmond metropolitan area (Fig. 9).

**Fig. 10:**
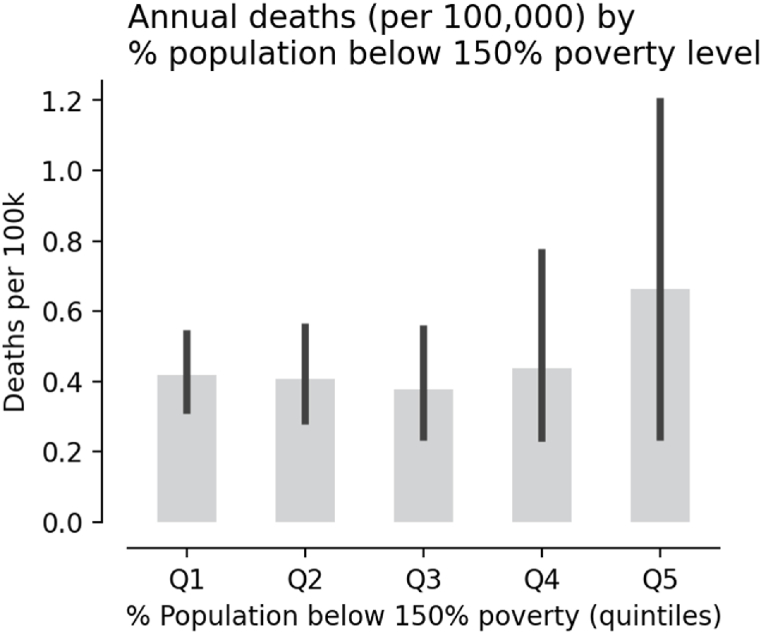
Avoided deaths due to power plant emissions by share of the population living below 150% the poverty level for each county.

Full decarbonization can also help reduce existing injustices related to pollutant exposure. We employ the Center for Disease Control's (CDC) Social Vulnerability Index (SOVI) data [41]. Specifically, we use the SOVI dataset's estimate of the share of the population in each county with income below 150% of the poverty line (EP_POV150), based on household income estimates from the 2016–2020 American Community Survey (ACS). We find that the benefits of avoided mortality are highest for those counties in the top quintile of EP_POV150. The value of EP_POV150 for these locations ranges between 30 and 50% of their total population. Mean annual avoided deaths across counties per 100,000 people in the bottom four quintiles were somewhat constant at a value of approximately 0.4 deaths per year, although the maximum value in the fourth quintile (Q4) reached up to 3.66 deaths per year (Fig. 10). Meanwhile, annual avoided deaths per 100,000 people in counties at the top quintile (Q5) was close to 1 deaths per year on average, with a maximum value of 6 deaths per year. On average, this represents a mortality rate increase of 50% in counties with most people living significantly below poverty across Virginia. These results are consistent with recent studies that found higher PM_2.5_ concentrations, as well as stronger links to mortality, in socioeconomically disadvantaged communities [43].

## 4 Discussion

Our analysis shows that completely phasing out of fossil fuel powered electricity production by 2045, as current plans aim and reflected in recent utility projections, will save the lives of Virginians, provide significant health-cost benefits, and reduce the burden of air quality on vulnerable communities and businesses. Results show that these benefits increase with decreases in fossil fuel emissions, with saved lives and reduced costs as early as 2025 under current plans. Finally, the results show that the benefits of a fossil fuel phase-out in the electric power sector will most benefit communities with a significant share of people living in poverty.

Limitations inherent to COBRA and simulation choices introduce uncertainty in results. The underlying air quality model is reduced in complexity in that it approximates air pollution transport and chemistry using simplified assumptions relative to a full complexity photochemical grid model. While the response surfaces cannot be observed, we rely on previous model evaluations of COBRA and similar reduced complexity models [44] against full complexity air quality models to establish general uncertainty bounds around the air quality response to emissions changes in our analysis. In a recent comparison of nationwide marginal damages between reduced complexity models, for example, Gilmore et al. (2019) [25] found differences across models of between 20 and 42% for elevated stationary sources (e.g., power plants), while Henneman et al. (2021) [26] found mean average errors at around 28% for low complexity models.

**Fig. 11:**
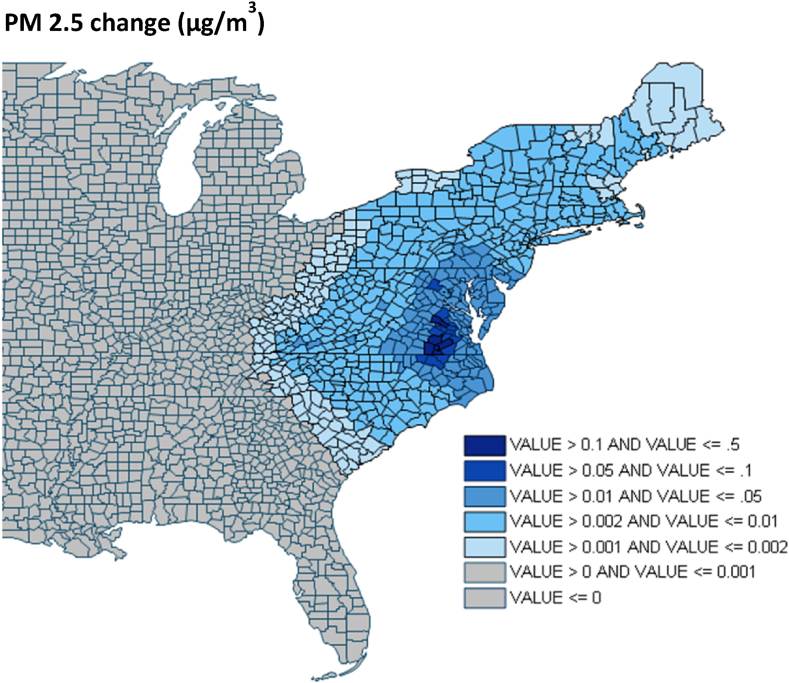
PM 2.5 concentration reductions across the eastern US from meeting VCEA goals.

The scenarios we simulated do not include additional externality of electricity generation besides air pollution emissions. One potentially large source of emissions may be due to transportation and sourcing of fossil fuels from sources to each power plant. A review by Steinmann et al. [45] showed that these upstream emissions range between 5 and 9% of coal-fired power's life cycle, although values vary significantly depending on fuel origin and method of extraction. Our analysis also does not prescribe any changes in emissions from sources outside the state of Virginia. This may add uncertainty to future emissions, particularly in counties close to state borders as nationwide fuel mix changes in the coming decades. However, we do estimate the impact of decarbonization within Virginia's border's outside state boundaries. While changes in PM_2.5_ are smaller as distance from the largest pollutant power plants increases, there are gains in nearby jurisdictions like Washington, D.C. and parts of North Carolina that are close in magnitude to those within the state itself (Fig. 11).

Uncertainties are also introduced in the formulation of dose-response functions based on previous studies. These formulations are based on observations of particular populations with particular distributions of age and vulnerabilities that can influence the development of health impact functions and their associated monetized benefits. To somewhat mitigate this limitation, we present, where available, low and high estimates of potential impacts in our simulation scenarios as a representation of a plausible range of benefits. The use of the SRM methos to compute changes in ambient concentrations of pollutants means that co-benefits are based on annual average. At the same time, the use of counties as the unit of analysis means that co-benefits in areas within counties with acute peaks (e.g., populations near power plants), may be underestimated.

## 5 Conclusion

This article presents an analysis of the health and health-cost benefits of transitioning from fossil fuels in utility power generation. Our work, which uses as an example the decarbonization pathway set by the VCEA and the state of Virginia, demonstrates the potential co-benefits of climate action. These co-benefits include avoided deaths, fewer hospital admissions from respiratory and cardiovascular issues, and fewer work loss days due to exposure to harmful pollutants like PM_2.5_. Not only do these co-benefits save lives and improve the health and livelihoods of people, they also present significant economic benefits, with the highest estimates showing nearly $350 million in avoided health and health-care costs per year once full shift towards non-carbon emitting generation is achieved in 2045.

These benefits are not evenly distributed throughout the state, and are largest where exposure is greatest. This includes counties with large populations located eastern Virginia, which include locations around the city of Richmond and Fairfax County (1.4 million population combined). Moreover, patterns of land use may have placed polluting power plants in disadvantaged communities throughout the state. The most disadvantaged communities have the most to gain. For instance, counties in the top quintile in percent population living below 150% of the national poverty line can expect to avoid 1.6 times more deaths than those in the bottom quintile.

Our analysis here shows that even when only accounting emissions directly associated with fossil-fuel combustion, there are significant benefits to human lives and health. Our work also shows that decarbonization of Virginia's power sector, traditionally viewed as only serving climate goals, can also serve communities currently suffering from disproportionate exposures. Future work can analyze the impact that decarbonization in the power sector can have on upstream emissions, as well as the impact on counties in neighboring states that may also benefit from decreased emissions in Virginia, which were not modeled in this analysis. More complex chemical transport models could be used to simulate impacts at the subcounty scale for specific power plants, which would allow for a detailed examination of environmental justice issues at the neighborhood scale. Finally, incorporating a pathway of specific power plant retirement and installation of renewable capacity would provide insight on the timing of specific benefits throughout the state, which we do not account for in here. Nevertheless, this work broadly highlights the transformative impacts of decarbonization of electric generation in addition to the benefits to climate impacts from reduced carbon emissions.

## Author contribution statement

conceived and designed the experiments; Luis Ortiz, Reilly Stiles, James Kinter, Edward Maibach. performed the experiments; Luis Ortiz, Reilly Stiles. analyzed and interpreted the data; Luis Ortiz, Reilly Stiles, Lucas Henneman, James Kinter, Jenna Krall, Edward Maibach, Benjamin Cash, Paul Bubbosh, Sophia Whitaker: wrote the paper; Luis Ortiz, Reilly Stiles, Lucas Henneman, James Kinter, Jenna Krall, Edward Maibach, Benjamin Cash, Sophia Whitaker.

## Data availability statement

Data will be made available on request.

## Declaration of competing interest

The authors declare that they have no known competing financial interests or personal relationships that could have appeared to influence the work reported in this paper.
